# Mechanism of traditional Chinese medicine in treating overactive bladder

**DOI:** 10.1007/s11255-022-03434-8

**Published:** 2022-12-07

**Authors:** Yuxiang Liao, Xin Du, Yuanbo Fu, Lu Liu, Jiangyan Wei, Qi An, Xuanzhi Luo, Fan Gao, Shuhan Jia, Ying Chang, Mengxi Guo, Huilin Liu

**Affiliations:** 1 Acupuncture and Moxibustion Department, Beijing Hospital of Traditional Chinese Medicine, Beijing Key Laboratory of Acupuncture Neuromodulation, Capital Medical University, Beijing, People’s Republic of China; 2 Beijing University of Chinese Medicine, Beijing, People’s Republic of China

**Keywords:** Lower urinary tract disease, Overactive Bladder, Traditional Chinese medicine, Therapy, Mechanism

## Abstract

Overactive bladder syndrome (OAB) has made increasing progress in mechanism and treatment research. Traditional Chinese medicine (TCM) is a common complementary therapy for OAB, and it has been found to be effective. However, the intervention mechanism of TCM in the treatment of OAB is still unclear. The aim of this review is to consolidate the current knowledge about the mechanism of TCM: acupuncture, moxibustion, herbs in treating OAB, and the animal models of OAB commonly used in TCM. Finally, we put forward the dilemma of TCM treatment of OAB and discussed the insufficiency and future direction of TCM treatment of OAB.

## Introduction

Overactive bladder syndrome (OAB) is defined as a complex of symptoms including urgency, frequency of urination, with or without urinary incontinence [1]. Urgency is the key symptom of OAB, defined as a sudden, intense urge to urinate that is difficult to postpone [2]. Epidemiological studies have shown that [3, 4], these symptoms affect 12–17% of adults in the community. Prevalence increases with age and is similar in men and women [5]. OAB imposes a huge economic burden on the health care system and the whole society. It is estimated that OAB has caused tens of billions of dollars in health budgets and broader economic losses in developed countries [6]. In addition to bringing an economic burden to the medical system, OAB will also cause serious damage to the quality of life of these patients [7]. The etiology of OAB is unknown, but it is known that various neurological and anatomical abnormalities of the lower urinary tract [8] as well as genetics, age, obesity, smoking, caffeine, etc., can cause or exacerbate OAB [9].

Current treatment options include behavioral interventions, medications (e.g., anticholinergic drugs, β-3 adrenergic receptor agonists), electrical nerve stimulation, and botulinum toxin A injections [10]. Anticholinergic and β-3 agonist therapy are the standard first-line pharmacological treatments for treating patients with OAB, but both have many corresponding side effects. For example, anticholinergic side effects include dry mouth, blurred vision, and constipation; β-3 agonist, while having a lower incidence of anticholinergic side effects, exhibit a higher risk of hypertension [11]. Therefore, it is urgent to find a convenient, more effective and less side effect treatment. However, TCM treatments such as acupuncture, herbal medicine and massage have been used in clinical practice for more than 2000 years and are considered as alternative therapies to treat various diseases [12], including treatment of symptoms such as frequent urination, urgency and incontinence in patients with OAB [13, 14]. The mechanism of TCM treatment of OAB is complex and the exact mechanism of different types of TCM treatment remains to be fully elucidated. In recent years, traditional Chinese medicine has achieved some success in the treatment of OAB. This paper reviews the clinical and mechanism of TCM treatment of OAB, hoping to bring new thinking for the treatment and research of OAB.

## Animal studies

At present, the understanding of OAB pathogenesis is still limited. Legal, ethical and moral constraints make it difficult to use human tissue for scientific research, and existing treatment guidelines are limited to alleviating symptoms. The application of the animal model in basic research is ethical and plays an important role in the connection between basic experiment and clinic [15]. Although there is no ideal animal model that can fully simulate OAB, we can use animal models of detrusor overactivity (DO) or bladder overactivity to further our understanding of the pathophysiology of OAB and to search for potential treatment options [16]. There are many kinds of OAB animal models, different modeling methods, different characteristics of models, different clinical diseases of OAB, and different application scope of models. These animal models can be categorized into two major types [15]: induced and transgenic/genetic models. Induced models can be further divided into the following categories: peripheral model, central model, induced hypersensitivity/inflammation model and bladder outlet obstruction (BOO) model. The peripheral model presented bladder dysfunction caused by direct impairment of peripheral bladder innerve, blood supply or metabolic state [17], such as OAB caused by feeding a high-fat diet in the hyperlipidemia model. Central models, on the other hand, present damage to the spinal cord, brainstem, or higher nerve centers, with rat T10 spinal cord transection being the most commonly used OAB modeling method for central nervous system injury [18], which can simulate bladder overactivity after human spinal cord injury and is also applicable to bladder C-fibers, bladder smooth muscle, and associated nerves. Hypersensitivity/inflammation models are induced by surgical or short infusion of chemically hazardous substances such as acetic acid, citric acid, capsaicin, cyclophosphamide (CYP) and alpha-bungarotoxin to induce hyper-sensitization of nociceptive afferent fibers in the bladder, which leads to bladder overactivity [19]. BOO model simulates bladder outlet obstruction in patients with an enlarged prostate by partially or completely ligating the urinary tract or bladder neck of rats [19]. It is also a reliable model for the study of lower urinary tract symptoms with etiological validity. Partial urinary tract obstruction in animals leads to some morphological and functional changes in the bladder and neural pathways similar to those pathological changes in humans [15]. Genetic and transgenic models, such as neuronal nitric oxide synthase and prostaglandin receptor knockout mouse models, are an advanced way to study the characteristics of urinary dysfunction [20]. In Table 1, we summarized the reports on the animal studies in OAB with TCM.

**Table 1:** A summary of relevant animal studies is presented

Animal models	Animals	Method	Intervention	Mechanism	Time	Journal	Author
Central model	Female SD rats	Spinal cord injury	Moxibustion	Inhibiting the M2/ATP/P2X3 pathway	2022	*Brain Res*	Wang et al.
Central model	Female SD rats	Spinal cord injury	Acupuncture	Inhibiting of TRPV1 channels	2021	*Journal of Practical Med*	Ning et al.
Central model	Female SD rats	Spinal cord injury	Acupuncture	Upregulating NGF and NT-3 expression in spinal cord tissue	2021	*Chin J of Rehabilitation Theory and Practice*	Ming et al.
Central model	Female SD rats	Spinal cord injury	Acupuncture	Inhibiting the expression of HCN channel	2020	*Ann Palliat Med*	Lu et al.
Central model	Aging SD female rats	Natural aging	Herbs	Increasing the sensitivity and expression of β_3_-AR	2017	*Exp Ther Med*	Xu et al.
Central model	Female SD rats	Spinal cord injury	Acupuncture	Inhibiting the apoptosis in spinal cord	2017	*Chin J of Rehabilitation Theory and Practice*	Xu et al.
Central model	Male SD rats	Fill in the brainstem within 0.5-M sodium acetate	Acupuncture	Changing the firing properties of bladder activity-related neurons in and around Barrington's nucleus	2012	*Neuroscience Research*	Wang et al.
Peripheral model	male C57BL/6 J mice	high-fat diet	herbs	Inhibition of purinergic neurotransmitter transmission	2019	*Front pharmacol*	Wang et al.
Hypersensitivity/inflammation model	Male Balb/c mice	Cisplatin-induced neurotoxicity	Acupuncture	Activating PACAP pathway to promote K^+^ channels	2021	*Front Immunol*	Li et al.
Hypersensitivity/inflammation model	Female SD rats	Intraperitoneal injection of CYP	Acupuncture	Inhibiting the ATP/P2X3 pathway	2020	*Evid-based compl alt*	Feng et al.
Hypersensitivity/inflammation model	Female Wistar rats	Intraperitoneal injection of CYP	Herbs	Inhibiting P2X3 receptor overexpression	2016	*J ethnopharmacol*	Lee et al.
Hypersensitivity/inflammation model	Female SD rats	0.1% acetic acid solution	Herbs	Inhibiting sensory transmission in C-fiber afferent pathways	2007	*J Urol*	Nishijima et al.
BOO model	Male SD rats	Oestrodial/testosterone-induced	Herbs	Inhibiting Ca^2+^ and ROCK signaling	2021	*J ethnopharmacol*	Miao et al.
BOO model	Female SD rats	Bladder neck ligation	Herbs	Enhancement of NO and its transduction pathway	2019	*BMC complem altern m*	Bae et al.
BOO model	male Wistar rats	Bladder neck ligation	Herbs	Promoting potassium channel expression	2017	*Evid-based compl alt*	Sun et al.
BOO model	Female SD rats	Bladder neck ligation	Herbs	Decreasing the expression of TRPV1	2015	*BMC complem altern m*	Lai et al.
BOO model	Female Wistar rats	Bladder neck ligation	Acupuncture	Regulating the excitability of bladder interstitial cells of Cajal	2013	*Evid-based compl alt*	Feng et al.
BOO model	Female SD rats	Bladder neck ligation	Herbs	Blocking L-type Ca^2+^ channel and activating BKCa channel	2013	*Eur Rev Med Pharmacol Sci*	Jiang et al.

Because of the complex pathogenesis of OAB and the subjective feeling of "urgency", there is no single model that can explain the complete mechanism of OAB in humans. The future development of OAB models should also take into account the complexity of the mechanism itself, fully include the advantages of the current models and reduce each other's shortcomings, so as to establish a more comprehensive model covering the multiple pathogeneses of OAB, to provide new references for future OAB mechanism research and interventions.

## Mechanisms of TCM in OAB

Acupuncture, moxibustion, herbal medicine, and massage, as classical TCM treatments, are regarded as an economical and safe alternative, and an increasing number of OAB patients may be willing to seek this treatment option with fewer side effects or less risks. There are several clinical trials by scholars in the United States, the United Kingdom and China, demonstrating the effectiveness and low side effects of acupuncture in the treatment of OAB [21–25]. Moxibustion is also a well-known non-invasive therapy that directly or indirectly stimulates acupoints through moxa to promote the recovery of many diseases, especially in chronic and refractory diseases [26]. Although not widely used in Western countries, moxibustion is a common method for symptom relief in Eastern regions such as China, Japan and Korea [27], and is easy for many OAB rehabilitation patients to perform on their own at home due to its efficacy, simplicity, and ease of use. This has recently attracted more attention from researchers [28]. Herbal therapy is based on syndrome differentiation to meet the individual needs of patients [29]. In recent years, a great deal of progress has been made in herbal medicine treatment of OAB, and some promising ingredients and TCM formulations have been found that can delay the progression of OAB, reduce the frequency and urgency of urination, and improve the recovery of bladder function [30]. Massage is also a simple, economical, safe and self-sufficient treatment and is one of the most popular treatments in complementary and alternative medicine for stress reduction, pain relief and other conditions [31]. It has been reported to be effective in alleviating the symptoms of OAB [32], even if massage is not as much evidence as acupuncture, herbal medicine, and recent exploration has yielded some evidence. In this article, the mechanism of TCM treatment of OAB is reviewed as follows:

### Improvement of bladder detrusor function

#### Effects of TCM on neurotransmitter pathways in OAB

The balance between the discharge and storage functions of the bladder requires a complex interplay of multiple neurotransmitters between the autonomic nerves (regulated by the sympathetic and parasympathetic nerves) and the somatic nerves (regulated by the pudendal nerves) [8]. Neurotransmitter signaling pathways mainly include cholinergic signaling pathways, purinergic signaling pathways, β adrenergic signaling pathways and adrenergic signaling pathways.

*Inhibition of cholinergic signaling pathways in OAB* In the cholinergic pathway [33–35], acetylcholine is a contractile neurotransmitter released by the sacral medullary parasympathetic post-ganglia fibers, which promotes detrusor contraction by mediating M receptor. M-Acetylcholine receptors can be divided into 5 subtypes, M1–M5. M2 and M3 receptors play an important role in detrusor contractile activity. M3 receptor plays a direct contractile role, while M2 receptor plays an indirect mediating role [36, 37]. Based on the cholinergic pathway, one study designed controlled experiments with M receptor agonists and M2 receptor blockers, and moxibustion significantly inhibited the expression of M2, but not M3, in the bladder detrusor muscle of neurogenic detrusor overactivity (NDO) rats [38]. Furthermore, a large number of experimental studies have also confirmed the regulatory effect of various Chinese herbal extracts on smooth muscle M receptor, which has been reviewed in detail [39].

*Inhibition of purinergic signaling pathways in OAB* Adenosine triphosphate (ATP) is a signaling molecule that regulates cellular processes and is an important factor in normal urine storage and urination. Bladder dysfunction such as OAB, lower urinary tract symptoms, bladder outlet obstruction and aging are associated with abnormal increase in ATP [40–42]. ATP in the bladder mainly comes from the urothelium [41], in the process of a full bladder, urothelium by mechanical force to stimulate the active release of ATP, then through acting on the afferent nerve endings P2X3 receptor activation micturition reflex, at the same time can also be applied to mesenchymal cells and detrusor cells P2X3 receptor on the surface of the cell membrane so as to promote the bladder contraction [43]. CYP-induced OAB rats showed increased ATP release in bladder urothelium, and the number of P2X3 receptor in bladder afferent nerve endings was significantly higher than that in normal rats [44]. Acupuncture can inhibit ATP overexpression and release in bladder epithelial cells of OAB rats and reduce the activation of the P2X3 receptor, thus it could effectively inhibit detrusor overactivity [45]. In the experiment of TCM formula Suo-Quan-Wan intervention in diabetic OAB model mice, the experiment showed that SQW could directly act on the bladder to improve the bladder function, rather than change the hyperglycemic status of mice and the mechanism may be that SQW inhibits ATP vesicle transport by down-regulating the expression levels of myosin Va and SLC17A9 in detrusor muscle [46]. Some researchers used moxibustion to intervene NDO rats, and the results showed that moxibustion could reduce the content of ATP in bladder mucosa, increase the content of NO in bladder mucous membrane, and reduce the expression of P2X3 receptor protein and mRNA in DO rats, thus improving bladder function [38]. In addition, Ba-Wei-Di-Huang-Wan, a Chinese medicine prescription, can improve CYP-induced persistent DO by inhibiting the overexpression of P2X2, P2X3, M2 and M3 receptor proteins in the mucosa, and the overexpression of M2 and M3 receptor proteins in detrusor muscle [47].

*Promoting nitrergic signaling pathways in OAB* In the urinary system, NO as a non-adrenergic, non-cholinergic inhibitory neurotransmitter is involved in the regulation of lower urinary tract function [48]. NO is synthesized by nitric oxide synthase, and NOS-positive nerve fibers are widely distributed in bladder and urethra tissues. Through the NO-cGMP signaling pathway, NO can effectively relax the lower urinary smooth muscle and reduce urethral pressure during normal urination, which is of great significance for maintaining normal urinary bladder storage and urination functions [49]. It is well established that reduced NO synthesis is closely associated with increased detrusor excitability due to a variety of causes [50]. Multiple studies [51, 52], have shown that electroacupuncture can promote the generation and secretion of nitrogen-energetic neurotransmitters in bladder tissue, thus improving bladder function. The TCM formula Wu-Zi-Yan-Zong-Wan (a mixture of berry extracts) can effectively treat bladder overactivity induced by partial urinary tract obstruction, and the formula shows anti-oxidant, anti-inflammatory, anti-physostigmine, as well as enhancing nitric oxide and its pathway [53]. The TCM formula Sang-Piao-Xiao-San also activates the PI3K/AKT-mediated NO-cGMP-PKG signaling pathway, upregulating NO production and thereby relaxing smooth muscle [54].

*Promoting adrenergic signaling pathways in OAB* Adrenergic β3 receptor (β_3_-AR) is the primary subtype of β-AR regulating detrusor relaxation in rats and humans, directly mediating detrusor relaxation [55]. β-AR receptor agonists such as isoproterenol can provoke detrusor diastolic muscle in mammals by enhancing cyclic adenosine monophosphate(cAMP) concentration [56]. This process includes: β-AR activates adenylate cyclase through excitatory G protein, and then catalyses ATP transformation to generate cAMP as a second messenger. As a second messenger, cAMP can activate PKA [57], thus coordinating the phosphorylation of smooth muscle contraction elements and increasing intracellular calcium reuptake and excretion. Stimulation of the calcium ion-activated K^+^ channel leads to membrane hyperpolarization, and other pathways ultimately lead to smooth muscle relaxation [56, 58, 59]. A study based on this pathway [60], observing the OAB model of natural aging rats, showed that the TCM formula Suo-Quan-Wan could regulate the contraction of the detrusor muscle by increasing the AC activity, cAMP and PKA content, and β3-AR receptor protein mRNA expression in detrusor cells.

**Fig. 1:**
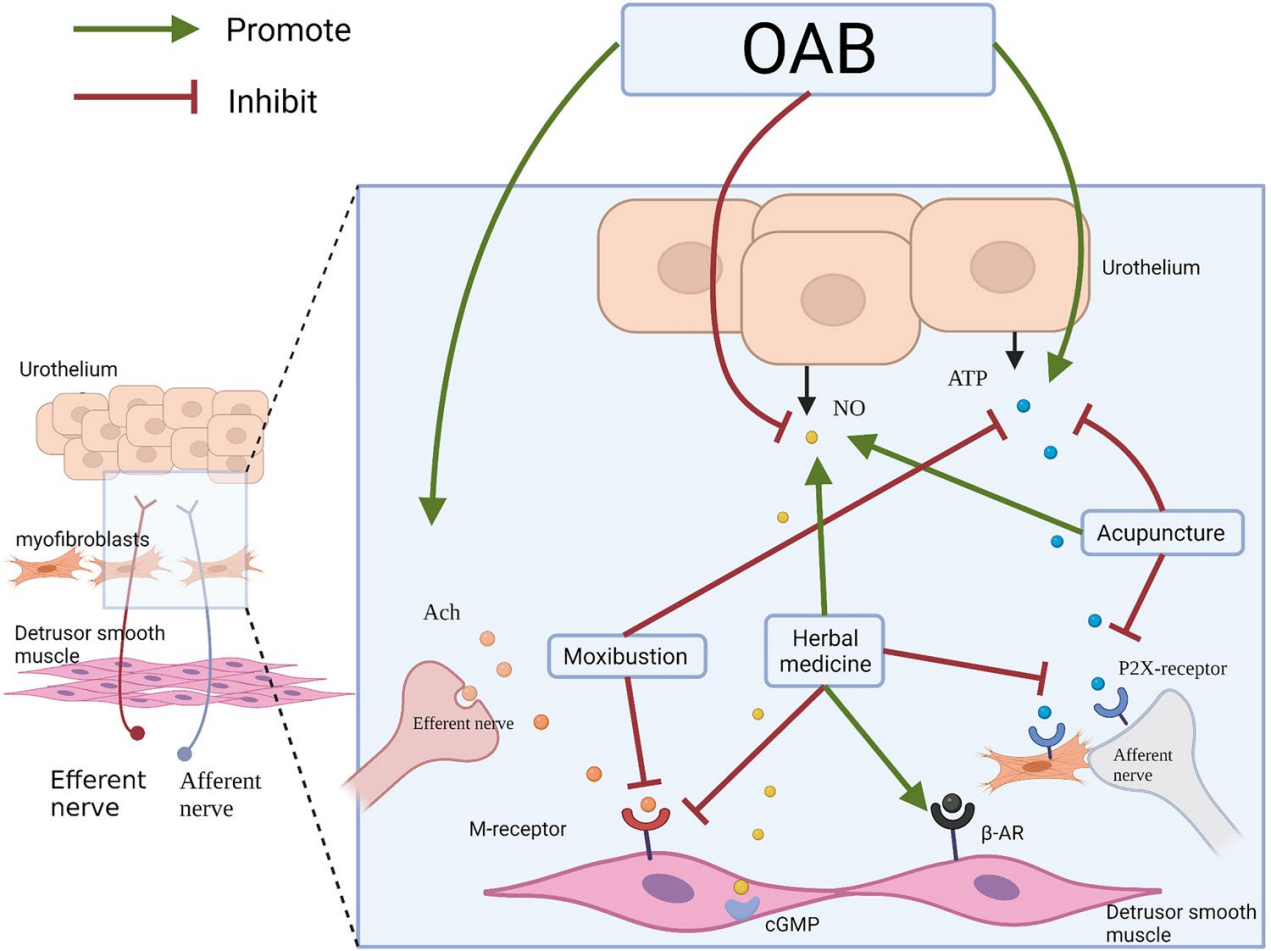
A model illustrating possible intervention mechanism in TCM affects the chemical interactions between urothelium, afferent nerves, efferent nerves, and fibroblasts in the urinary bladder. Acupuncture and moxibustion can promote the synthesis and release of NO, reduce the synthesis and release of ATP, down-regulate the expression of P2X receptor protein, and inhibit the excessive contraction of the detrusor muscle. Herb medicine can participate in the regulation of multiple neurotransmitters, such as Suo-Quan-Wan can inhibit the expression of the M receptor and P2X receptor, and up-regulate the expression of the adrenergic receptor. Wu-Zi-Yan-Zong-wan can enhance the role of nitric oxide and its transduction pathway

In summary, prescriptions composed of the same herbs can show different targets. Acupuncture at the same acupoint can also produce various effects. This may be a multi-target display of TCM. As shown in Fig. 1, acupuncture, moxibustion and herbal medicine affect various neurotransmitter pathways. It is noteworthy that the role of urothelium has attracted increasing attention, emphasizing an afferent "urotheliogenic" hypothesis that urgency begins with urothelium [61]. In short, the possibility of bladder dysfunction was elucidated through the perception of mechanical and chemical abnormalities in the urothelium. These studies provide new ideas for exploring the mechanism of TCM. At present, the research on the mechanism of TCM in treating OAB is mostly analyzed from a single point of view, but the multi-structure and the multi-link relationship has not been analyzed and discussed, which can not fully reflect the two-way regulation and multi-target characteristics of TCM. The neurotransmitter effects of TCM on OAB need further study.

#### Effects of TCM on ion channel pathways in OAB

In addition to the neurotransmitter pathways, the ion channel pathways are one of the main mechanisms that regulate the coordination of contraction and relaxation of bladder smooth muscle cells [62], including voltage-gated calcium channels (Ca_v_) and transient receptor potential (TRP) channels for enhanced excitation of the detrusor muscle, and K^+^ channels for reduced excitation [63].

*Ca*^*2*+^ *channel* The dynamic interaction between bladder smooth muscle ion channels determines the overall level of Ca_v_ channel activity. The net influx of calcium through the Ca_v_ channel increases the intracellular calcium concentration, which triggers bladder smooth muscle contraction [63]. Under pathological conditions, high Ca^2+^ concentration in detrusor muscle cells is one of the important factors leading to detrusor muscle overactivity [64]. Berberine, derived from herbal Coptis chinensis Franch, improves bladder function by significantly inhibiting intracellular Ca^2+^ levels and ROCK-mediated contraction of detrusor cells [65]. Similarly, rhynchophylline, derived from the Uncaria plant, can inhibit the contraction of rat bladder strips by blocking L-type calcium channels and activating big-conductance calcium-activated potassium channels [66].

*K*^+^ *channel* Several families of K^+^ channels expressed and functional in bladder smooth muscle include voltage-gated K^+^ channels, Ca^2+^-activated K^+^ channels, inward-rectifying ATP-sensitive K^+^ channels, and two-poredomain K channels [67]. In general, the opening of the K^+^ channel causes hyperpolarization of the cell membrane, limiting the flow of calcium ions through the L-type Ca_v_ channel and causing relaxation of the bladder smooth muscle. Conversely, inhibition of K^+^ channels in detrusor smooth muscle cells leads to membrane depolarization and activation and opening of L-type Ca_v_ channels, leading to detrusor smooth muscle contraction [68]. The advantage of K^+^ channel opening agent in the treatment of OAB is that it can reduce the involuntary contraction during urination storage and the contractile activity caused by external stimulation, without affecting the bladder pressure during urination [69]. There is still a lack of K ^+^ channel openers with bladder specificity, and in the future, the use of bladder-specific K^+^ channel openers may replace current first-line pharmacotherapy [67]. There is increasing evidence that TCM can inhibit the involuntary contraction of the bladder urine storage period without affecting the contraction of the detrusor muscle during the bladder urination period, which is similar to the effect of K^+^ channel openers [38, 45]. Therefore, we believe that K^+^ channel may be one of the important targets for TCM treatment of OAB. For example, abnormal expression of potassium channels was found in detrusor muscle in BOO-related OAB model rats. Xian-jia-tang, a Traditional Chinese medicine decoction, could significantly inhibit the expression of K_v2.1_ and K_v1.5_ in detrusor muscle, but increased the expression of BK, SK_2/3_ and K_ATP_, suggesting that the effect of Xian-jia-tang on BOO rats was related to the K^+^ channel in detrusor muscle [70]. In addition, acupuncture could be upregulated, pituitary adenylate cyclase-activating polypeptide [71], and then cAMP-activated PKA, promoting phosphorylation of K^+^ channel and hyperpolarization of the cell membrane. This indirectly inhibits the activity of L-type voltage-dependent calcium channels on the cell membrane, prevents extracellular Ca^2+^ from entering muscle cells, reduces intracellular Ca^2+^ concentration and promotes the relaxation of smooth muscle cells [72].

*TRP channel* Transient receptor potential channels are non-selective cation channels, including TRPV1, TRPV2, TRPV4, TRPM8, and TRPA1, and act as sensors for stretch and/or chemical stimulation to convey the sensation of bladder dilation [73, 74]. In the urinary system, TRPV1 is the most studied channel [74], which is expressed in primary sensory afferent fibers, urinary tract epithelium and smooth muscle cells and promotes afferent excitation during bladder filling [75]. Desensitization of this channel with TRPV1 agonists is often used clinically to treat OAB, and its clinical use has been reviewed in detail elsewhere [76–78]. Animal experimental studies showed that TRPV1 protein expression in the bladder tissue of NDO was significantly higher than that in the sham-operated group, and TRPV1 protein expression levels in the bladder tissue of rats in both the electroacupuncture and inhibitor groups were significantly lower compared with the model group [79]. Suo-Quan-Wan can also promote the recovery of bladder function by regulating TRPV1 expression [80].

*HCN channel* Hyperpolarization-activated Cyclic Nucleotide-gated channels (HCN) in bladder interstitial cells of Cajal (ICC) are important and special ion channels in bladder ICCs [81]. At the same time, HCN is the structural basis for the generation of overactivated positive ion flow currents, and an increase or decrease in its protein expression directly causes excitability in pacemaker cells, which may have a critical role in the maintenance and regulation of bladder cell excitability [81]. In the neurogenic OAB rat model, the expression of HCN channel in ICCs was significantly up-regulated after spinal cord injury, while electroacupuncture at "Ci-Liao" and "Hui-Yang" acupoints inhibited the expression of HCN channel [82]. In addition, experiments with acupuncture intervention in BOO-related OAB model rats showed [83] that HCN2 mRNA and protein expression was upregulated in the bladder of OAB rats, and acupuncture reversed this rise; ICC results in the bladder of OAB rats cultured in vitro showed higher intracellular Ca^2+^ concentrations than in normal control rats, and Ca^2+^ concentrations decreased after acupuncture treatment.

The importance of ion channels in the excitability and contractility of detrusor suggests that defects, alterations, or mutations in ion channels may lead to certain forms of bladder lesions, also known as channelopathies [84]. At present, there have been many reports that TCM can treat OAB through ion channels, but these experiments have not yet described the upstream mechanism of regulating ion channels of TCM in detail, which needs further study.

### Improvement of abnormal bladder nerve signals in OAB

#### Inhibition of C-fiber activity

In recent years, the role of afferent nerve fibers in OAB has been paid more and more attention [85]. The main nerves controlling the bladder are the hypogastric nerve, pudendal nerve and pelvic nerve [86]. Among them, the pelvic nerve is parasympathetic and is the most important nerve involved in bladder function, and most of the afferent fibers from the bladder enter the sacral medullary voiding center through the pelvic nerve [87, 88]. The bladder afferent nerves, which can be classified as myelinated sensory A-delta fibers and unmyelinated sensory C-fibers, respond to mechanical and chemical stimuli and then transmit information to the central nervous system about bladder volume and the chemical environment inside the bladder [89]. C-fibers hyperactivity is one of the key pathological mechanisms of OAB [90]. In OAB, changes in peripheral afferent receptors may result from increased release of neurotrophic factors (such as nerve growth factor) in the spinal cord or bladder, resulting in silenced C-fiber sensitization and increased afferent impulses [91, 92]. Some scholars believe that [93] when the needle tip is close to the anterior branch of the second sacral nerve at the deep point of “Zhong-Liao”, it can stimulate the sacral nerve, thus inhibit the excitement of the afferent nerve of the bladder. This process is similar to the regulation of the sacral nerve [94, 95]. It has also been reported that 5-hydroxytryptamine induces hyperexcitability of the C-fiber afferent pathway, leading to pain or bladder overactivity in the presence of mucosal inflammation [96]. Nishijima found a decrease in plasma 5-hydroxytryptamine levels in hypersensitivity OAB model rats after treatment with the TCM formula Ji-Sheng-Shen-Qi-Wan, suggesting that it can inhibit sensory transmission in the C-fiber afferent pathway and maintain the balance between the sympathetic and parasympathetic nervous systems at a lower level to suppress bladder activity [97].

#### Inhibition of the release of substance P from the afferent nervous system

Substance P (SP) is a sensory neuropeptide found in the afferent nervous system of mammals and innervates smooth muscles such as the detrusor muscle [98]. Once released from the bladder afferent nerve, SP is involved in the mechanoreceptor-mediated micturition reflex [99]. Studies have shown that bladder biopsies from patients with idiopathic detrusor overactivity show a significant increase in fibers containing SP [100], suggesting that SP is closely associated with the occurrence of OAB. Tsai WH showed [101], that excessive SP stimulation enhances the voiding reflex through activation of neurokinin type 1 receptors and increases the release of reactive oxygen species from intercellular adhesion molecule-1 mediated adhesion and infiltration of inflammatory cells, leading to chronic bladder inflammation and hyperreflexia. The Chinese herbal compound Ba-Wei-Di-Huang-Wan and its active ingredient strychnine may ameliorate OAB by inhibiting SP-induced bladder afferent signaling, intercellular adhesion molecule-1 expression, and reactive oxygen species counts.

### Improvement of neuronal activity in the brain in OAB

Advanced voiding centers include the pontine micturition center (PMC) and periaqueductal gray (PAG) [102]. The PAG receives afferents from the lumbosacral medulla and is directly connected to the PMC, which controls voiding via the efferent sacral parasympathetic nucleus [103]. Studies have shown that psychological stress or increased sympathetic activity leads to central sensitization, which in turn leads to OAB [104]. In the pathogenesis of central sensitization, peripheral nerves usually function normally, but the central neuronal function is altered [105]. These highly sensitive central system neurons show reduced thresholds, greater evoked responses, increased receptive field sizes and ongoing stimulus-independent activity [106]. Wang and his colleagues showed [107] that acupuncture at the sacrum alters the firing patterns of PMCs and their surrounding neurons related to bladder activity; further application of a GABA receptor antagonist (bicuculline) can block the inhibition of bladder activity, suggesting that acupuncture can reduce DO by modulating the GABA receptor system mediating, and thus regulating, the firing of PAG and PMC neurons.

### Improvement of the spinal urinary center function in OAB

The spinal cord plays an important role in bladder afferent and efferent signal transduction. The sensory signals during the bladder filling travel upward through the afferent nerves to the dorsal root ganglion of the spinal cord in the lumbosacral segment, where the posterior horn of the spinal cord integrates the sensory information and sends it to the higher voiding center. Therefore, the structure and function of the spinal cord is important in regulating the maintenance of normal bladder voiding [89]. After the injury above the sacral spinal cord, the inhibitory and excitatory controls from the high micturition center on the sacral micturition center are lost, and a new C-fiber-dependent reflex arc is formed in the sacral spinal cord, thus causing bladder overactivity [108]. A Studies showed that electroacupuncture significantly inhibited the expression of apoptotic protein Caspase-3 in the spinal cord tissues of rats; further studies showed that electroacupuncture significantly inhibited cytochrome C and apoptotic protease-activating factor-1 and activated Caspase-9 protein, reduced the rate of spinal cord apoptosis, alleviated secondary spinal cord apoptosis, promoted spinal cord functional remodeling, alleviated secondary spinal cord injury, and regulated innervation of the bladder and pelvic floor [109]. In recent years, it has been proposed that the mechanism of OAB action after spinal cord injury treated with TCM may involve neuro-endocrine network regulation in addition to inhibition of spinal cord cell apoptosis [110]. For example, several studies have shown that acupuncture can increase the protein and mRNA content of endogenous nerve growth factor, neurotrophin 3, and tyrosine kinase receptor A in the spinal cord tissue of DO rats after supra-sacral spinal cord injury, promote the repair of injured nerves and recovery of nerve function and improve bladder storage capacity [111–113]. In addition, it should be noted that the whole bladder is rich in nerve growth factors (NGF) that maintain nerve growth and function in the bladder and inhibit apoptosis of nerve cells, which makes the bladder more sensitive. In the DO rat model of spinal cord injury, NGF was expressed in the epithelial and detrusor muscles of the bladder in both groups, and the expression of NGF was significantly higher in the bladder of the model group [114]. Increased NGF expression in the bladder can induce bladder overactivity [115]. Several meta-analyses have also shown a strong relationship between urinary NGF levels and both the diagnosis and treatment of OAB [116]. Therefore, NGF, a nerve growth factor in urine, may serve as a potential biological marker for the diagnostic treatment of OAB [117]. As science advances, we believe that in the future, NGF as a conveniently detectable biomarker can provide reliable evidence for the clinical treatment of OAB after spinal cord injury in TCM.

## Dilemma in the research of TCM in OAB

**Fig. 2:**
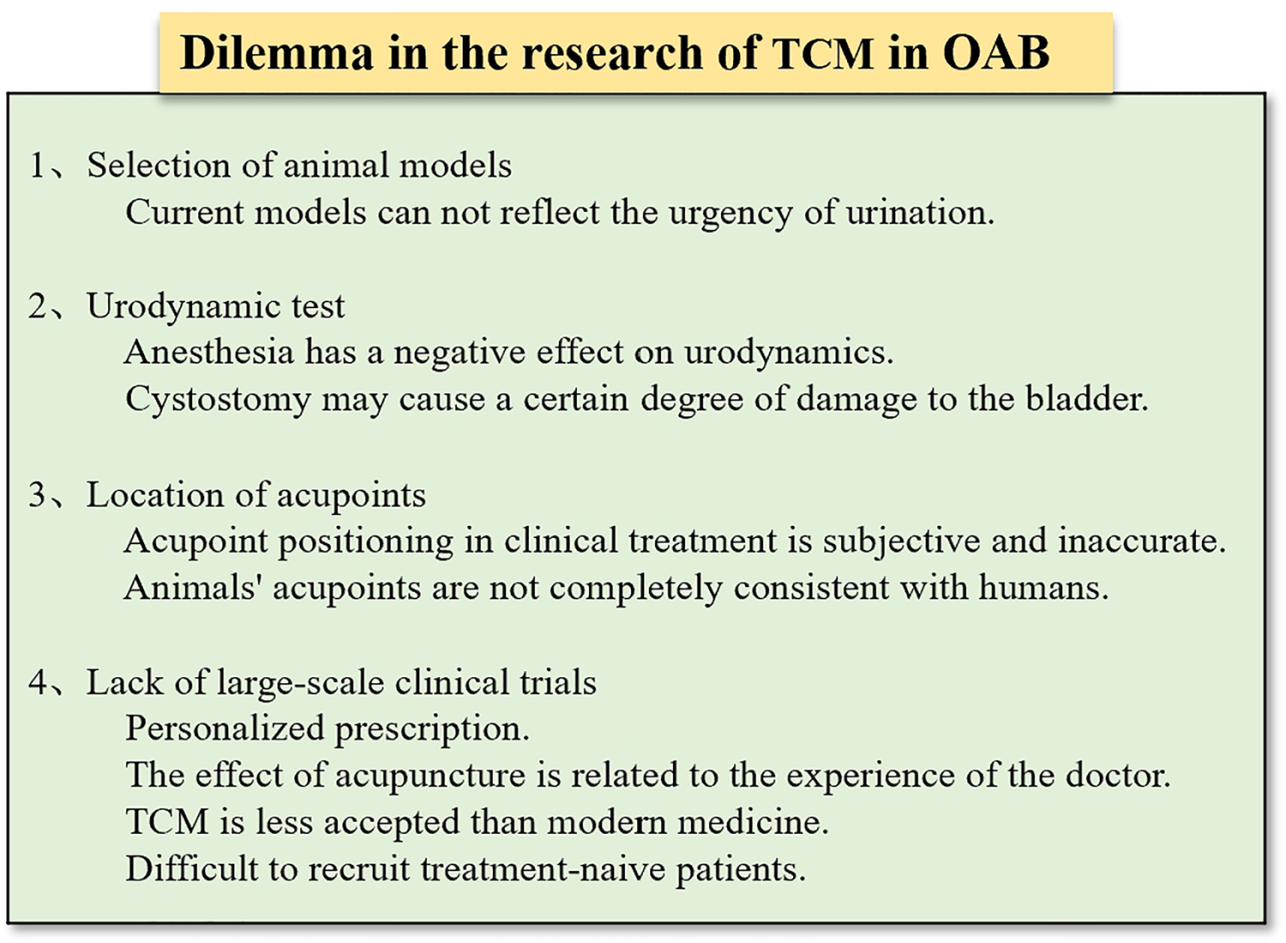
Dilemma in the research of TCM in OAB

In recent years, TCM treatment of OAB has made some progress, but still faces many difficulties, as shown in Fig. 2. First, the selection of animal models. There are many kinds of OAB animal models, and the application scope of OAB models is also different, which has been reviewed previously. Therefore, how to choose a more suitable model for TCM research needs further exploration, and the current research is mostly from a single angle of analysis, there is no multi-structure, multi-link relationship series analysis. Second, the test of the model—urodynamic detection. Animal models for OAB pathophysiological studies have been successfully established in several species. However, OAB is a clinical diagnosis, and a subconscious sense of urgency is the key to the diagnosis. Different from the urodynamic diagnosis of DO. There is an overlap between urodynamically defined DO and subjectively reported OAB. Not all patients with OAB have DO and DO patients DO not always have a sense of urgency [118]. Therefore, it is technically impossible to establish a specific animal model of OAB. Although the symptoms of urgent urine in animals cannot be quantified, we can substitute them by objective observation of the measurement of bladder pressure in DO. This is why most OAB experimental models are built on DO, and urine flow mechanics is the gold standard to judge the success of model construction. There are two commonly used methods for bladder measurement in animals: manometry in an awake state and manometry in anaesthetic state. Awake bladder manometry is the most widely used method of bladder manometry at present. This method is operated on animals with cystostomy, and bladder manometry is carried out in their awake state. This manometry method is not affected by narcotic drugs and is most consistent with the physiological conditions of rats [119]. However, after cystostomy, there is certain damage to the bladder, which will also affect bladder function [120]. More difficult, we usually conduct a urodynamic test first to confirm the successful establishment of the model, and then start intervention. After cystostomy, moxibustion and acupuncture are difficult to implement. Although bladder manometry under anesthesia is highly controlled and non-invasive to the bladder, the influence of anesthetics on bladder function cannot be ruled out, which may have an impact on the evaluation of the efficacy of an overactive bladder. Therefore, how to eliminate the effect of anesthetics on bladder function and increase the controllability of awake manometry in rats may be an urgent problem to be solved. Third, the accurate location of acupoints plays an important role in acupuncture and moxibustion clinic. However, due to the differences in individual patients and doctors' experience, the location of acupoints in clinical treatment has certain subjectivity and inaccuracy, which affects the curative effect of treatment to varying degrees. In addition, in animal studies, there is no unified standard for animal acupoint location, and there is a big difference between animal acupoint location and human acupoint location. Fourth, at present, there are few large-scale clinical trials to prove the effectiveness of TCM. As mentioned above, the therapeutic effect of acupuncture is closely related to the experience of the physician. To achieve the best results, doctors will adjust the composition of drugs or the collocation of acupoints during the follow-up in real time, which often leads to different prescriptions for each person [121]. Patient acceptance of TCM is lower than that of modern medicine, which makes it difficult to recruit treatment-naive patients. Therefore, these realities present difficulties in the design and operation of clinical or basic trials, and further research is needed to understand the long-term efficacy and mechanism of action of this intervention.

## Conclusions

In this article, we have summarized the mechanism of Traditional Chinese Medicine in treating with OAB, and summarized the current OAB animal models in TCM treatment. According to the pathogenesis of OAB, we infer that the mechanism of TCM in treating OAB may be to improve the release of abnormal neurotransmitters in urinary tract epithelial cells and the incoming signal of the bladder based on "urotheliogenic" hypothesis. Finally, we point out the development dilemma and prospect of Chinese medicine in OAB. These studies provide a theoretical basis for the clinical treatment of OAB with TCM. TCM may become an effective treatment alternative, with simple operation, low cost and few side effects, although it needs further well-designed research to determine the optimal regimen.

## Data Availability

The datasets generated during or analysed during the current study are available from the corresponding author on reasonable request.
